# A Prognostic Model Based on the Immune-related Genes in Colon Adenocarcinoma

**DOI:** 10.7150/ijms.45813

**Published:** 2020-07-19

**Authors:** Yuan-Lin Sun, Yang Zhang, Yu-Chen Guo, Zi-Hao Yang, Yue-Chao Xu

**Affiliations:** Department of Gastrointestinal Surgery, The First Hospital, Jilin University, Changchun 130021, Jilin Province, China.

**Keywords:** immune-related genes (IRGs), differential expressed analysis, prognostic model, Cox regression analysis, colon adenocarcinoma (COAD)

## Abstract

**Background:** Immune-related genes (IRGs) are critically involved in the tumor microenvironment (TME) of colon adenocarcinoma (COAD). Here, the study was mainly designed to establish a prognostic model of IRGs to predict the survival of COAD patients.

**Methods:** The Cancer Genome Atlas (TCGA), Immunology Database and Analysis Portal (ImmPort) database, and Cistrome database were utilized for extracting data regarding the expression of immune gene- and tumor-related transcription factors (TFs), aimed at the identification of differentially expressed genes (DEGs), differentially expressed IRGs (DEIRGs), and differentially expressed TFs (DETFs). Univariate Cox regression analysis was subsequently performed for the acquisition of prognosis-related IRGs, followed by establishment of TF regulatory network for uncovering the possible molecular regulatory association in COAD. Subsequently, multivariate Cox regression analysis was conducted to further determine the role of prognosis-related IRGs for prognostic prediction in COAD. Finally, the feasibility of a prognostic model with immunocytes was explored by immunocyte infiltration analysis.

**Results:** A total of 2450 DEGs, 8 DETFs, and 79 DEIRGs were extracted from the corresponding databases. Univariate Cox regression analysis revealed 11 prognosis-related IRGs, followed by establishment of a regulatory network on prognosis-related IRGs at transcriptional levels. Functionally, IRG GLP2R was negatively modulated by TF MYH11, whereas IRG TDGF1 was positively modulated by TF TFAP2A. Multivariate Cox regression analysis was subsequently performed to establish a prognostic model on the basis of seven prognosis-related IRGs (GLP2R, ESM1, TDGF1, SLC10A2, INHBA, STC2, and CXCL1). Moreover, correlation analysis of immunocyte infiltration also revealed that the seven-IRG prognostic model was positively associated with five types of immunocytes (dendritic cell, macrophage, CD4 T cell, CD8 T cell, and neutrophil), which may directly reflect tumor immune state in COAD.

**Conclusions:** Our present findings indicate that the prognostic model based on prognosis-related IRGs plays a crucial role in the clinical supervision and prognostic prediction of COAD patients at both molecular and cellular levels.

## Introduction

Colorectal cancer (CRC) is one of the primary lethal malignancies which can be divided into colon cancer and rectal cancer based on the corresponding primary tumor sites 1. The incidence cases of CRC are estimated to exceed 2.2 million and the death cases are predicted to exceed 1.1 million deaths by 2030 2. Colon adenocarcinoma (COAD) is considered as the most common pathological type of CRC 3. The incidence and mortality of COAD accounts for 6.1% and 5.8%, respectively, ranking the fourth and fifth among all types of new cancer cases 4. Although the diverse types of advanced therapies, including surgery, adjuvant radiation therapy or chemotherapy, and targeted molecular therapy, are currently used to treat CRC, the poor prognosis and survival of patients demand prompt solutions due to delayed diagnosis and adverse drug effects 5. In consideration of the dilemma, it is urgent to explore new biomarkers, which might exert an impact on the prognosis of COAD.

At present, immunotherapy is wisely applied in the individualized treatment in a variety of tumors. Of note, interferons (IFNs) have long been demonstrated to play diverse roles, including antimicrobial and antiviral response, cell cycle progression, apoptosis and mediators of other cytokines 6-8. More recently, James et al. have revolutionarily concluded that blocking CTLA-4 would prime T-cells to attack cancer cells 9, and Salem M et al. have revealed the removal of cancer cells by knocking out GARP of Treg cells 10. Piero et al. have estimated that CDX2 could be recognized as a biomarker for malignant tumors in clinical surveillance and prognosis due to the fact that patients with stage II and stage III colon cancer and a lack of CDX2 suffered neoplasm recurrence and subsequent death 11. These findings have illustrated the therapeutic importance of immune systems in COAD. Recently, Li et al. and Peng et al have expounded the prognostic value of immune-related genes (IRGs) to non-small cell lung cancer and papillary thyroid cancer, respectively 12, 13. However, it remains unknown of the clinical correlation and prognostic evaluation of IRGs in COAD.

The present study was designed to explore the potential correlation between the clinical prognosis and immune-related genes (IRGs), which were molecular biomarkers that can be further applied to individualized and targeted therapies. In particular, Cox regression analysis was performed to construct an IRGs-based prognostic model. The visual regulatory network formed by DETFs and prognosis-related IRGs demonstrated the potential mechanisms at a molecular level. Additionally, immune infiltration analysis concerning seven-IRG prognostic model as well as immunocyte accumulation might shed new light on the function of immunocytes for prognostic prediction in COAD.

## Methods

### Patient samples and data collection

The transcriptome RNA-sequencing data were retrieved and downloaded from The Cancer Genome Atlas (TCGA) data portal (https://portal.gdc.cancer.gov/); a total of 473 tumor tissues and 41 healthy tissues were included. The data on IRGs were downloaded from the Immunology Database and Analysis Portal (ImmPort) (https://www.immport.org/) 14. Moreover, the cancer transcriptional genes data were available in Cistrome Project (http://www.cistrome.org/).

### Functional enrichment analysis for DEIRGs

To integrate and analyze the IRGs among transcriptome RNA-sequencing data and IRGs, the cancer transcriptional genes data, and the “limma package” in R software 15 was used for data management. Subsequently, DEIRGs were screened, and the cut-off value assigned for false discovery rate (FDR) <0.05 and |log_2_ fold change| (|logFC|) >1. DEIRGs were used to elucidate the underlying molecular mechanisms based on the findings from Database for Annotation, Visualization and Integrated Discovery (DAVID), Gene Ontology (GO), and Kyoto Encyclopedia of Genes and Genomes (KEGG) pathway analysis. For GO analysis conducted using “GO plot packages” (https://cran.rproject.org/web/packages/GOplot/citation.html) 16, GOCircle and GOChord graphs were obtained on the basis of a precondition and by setting a *P* <0.05 to indicate statistical significance.

### Prognosis-related IRGs and DETFs

After incorporating overall survival (OS) and DEIRGs of patients using Perl language, we performed univariate Cox regression analysis for the preliminary screening of prognosis-related IRGs based on a *P* <0.05. Afterwards, prognosis-related IRGs were categorized into high-risk prognosis-related IRGs (HR>1) and low-risk IRGs (HR<1) based on hazard ratios (HRs) derived from univariate analysis. TFs bound with specific DNA sequences and are thereby critically involved in the direct regulation of gene expression. In this study, relevant TFs were acquired from Cistrome database (http://www.cistrome.org/), which is an online tool, and from TCGA by integrating chromatin profiling data and cancer genomics data, respectively, which in turn facilitated the exploration of regulatory correlations of TFs with cancer transcriptomes 17. For further investigating the association of DETFs, which were extracted from the Cistrome database, with prognosis-related IRGs obtained on the basis of univariate Cox regression analysis, the regulatory network was constructed 18, 19 on the basis of a co-expression analysis according to Cor filter >0.04 and a *P* value filter <0.001 and the TF regulatory network was visualized using Cytoscape 3.7.2.

### Prognostic model construction

Multivariate Cox regression analysis was further conducted on prognosis-related IRGs that were obtained based on univariate analysis, aiming at establishing a prognostic model and subsequently calculating the riskScore. Moreover, COAD patients were divided into two groups based on their riskScore, namely the low-risk and high-risk group. Kaplan-Meier (K-M) survival analysis with survival R packages could be used for obtaining the survivorship curve, which revealed the difference in terms of survival between the low-risk and high-risk group by demonstrating their respective survival periods and their corresponding survival rates. Furthermore, the risk plot R package was used to plot the risk curve to reveal the difference in survival periods between the low-risk and high-risk group. And the receiver operating characteristic (ROC) curve along with the area under the curve (AUC) could be obtained by analyzing the prognostic value of prognostic model.

### The correlation between the prognostic model and immunocytes

Tumor Immune Estimation Resource (TIMER) (https://cistrome.shinyapps.io/timer/), a publicly available resource for analyzing and visualizing the abundance of tumor-infiltrating immune cells, includes 10,897 samples encompassing 32 types of cancer identified from TCGA and can be used for estimating the abundance of six TIIC subsets (CD4 T cells, CD8 T cells, B cells, neutrophils, macrophages, and dendritic cells) 20. In this study, we carried out immunocyte infiltration analysis with the Immune Estimation file retrieved from TIMER to explore the underlying correlation between the prognostic model and immunocytes.

### Further verification on IRGs in the prognostic model

Oncomine (http://www.oncomine.org) 21 and TIMER (https://cistrome.shinyapps.io/timer/) 22 were both employed for further verifying differences in the expression of IRGs between tumor tissues and healthy tissues in the prognostic model. Moreover, Human Protein Atlas (HPA) (https://www.proteinatlas.org/) 23 was used to extract immunochemical images, which showed the distribution and protein expression of IRGs in the prognostic model.

## Results

### Identification of DEGs, DEIRGs, and DETFs

**Figure 1:**
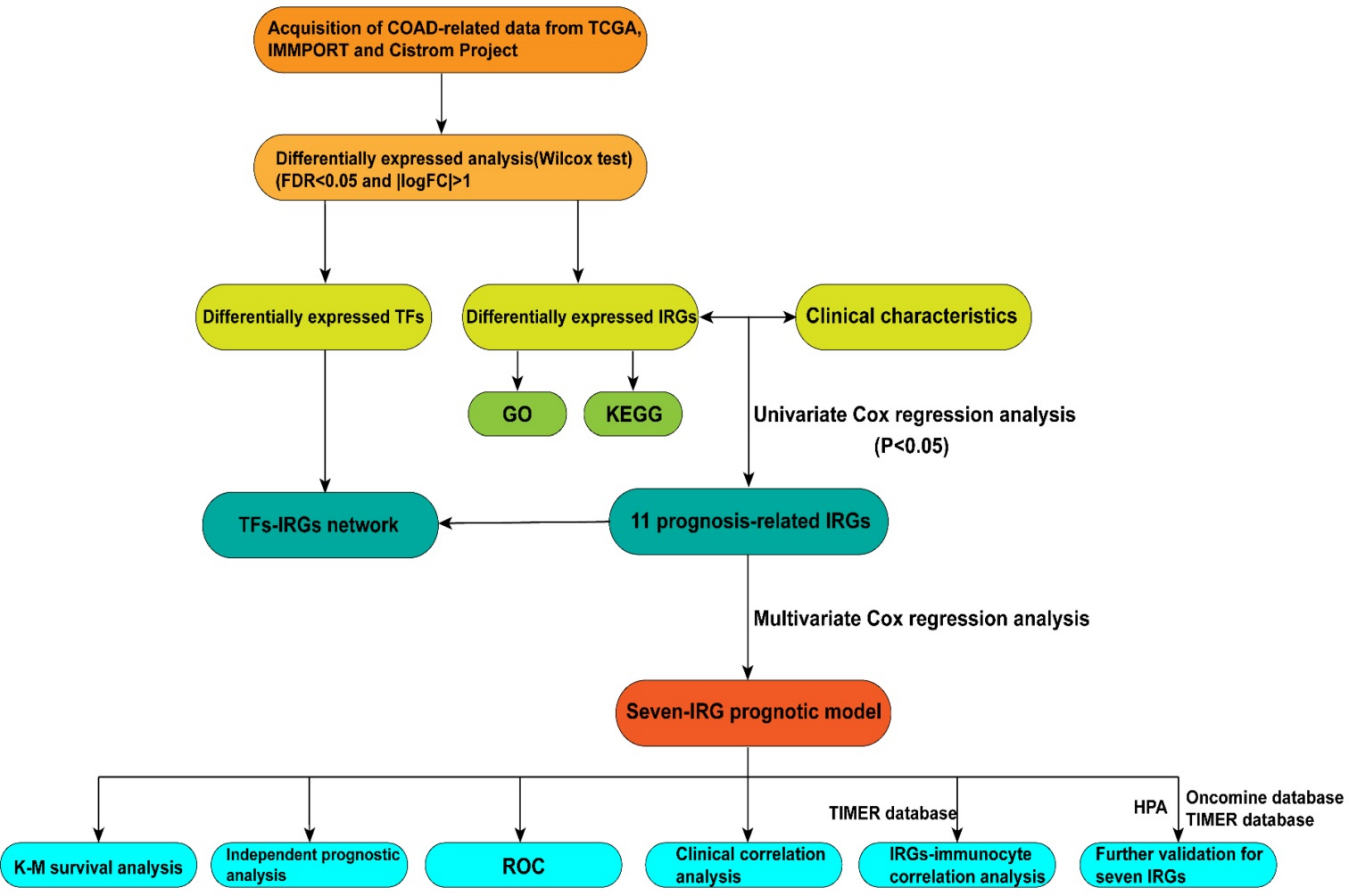
Flow diagram of the study.

**Figure 2:**
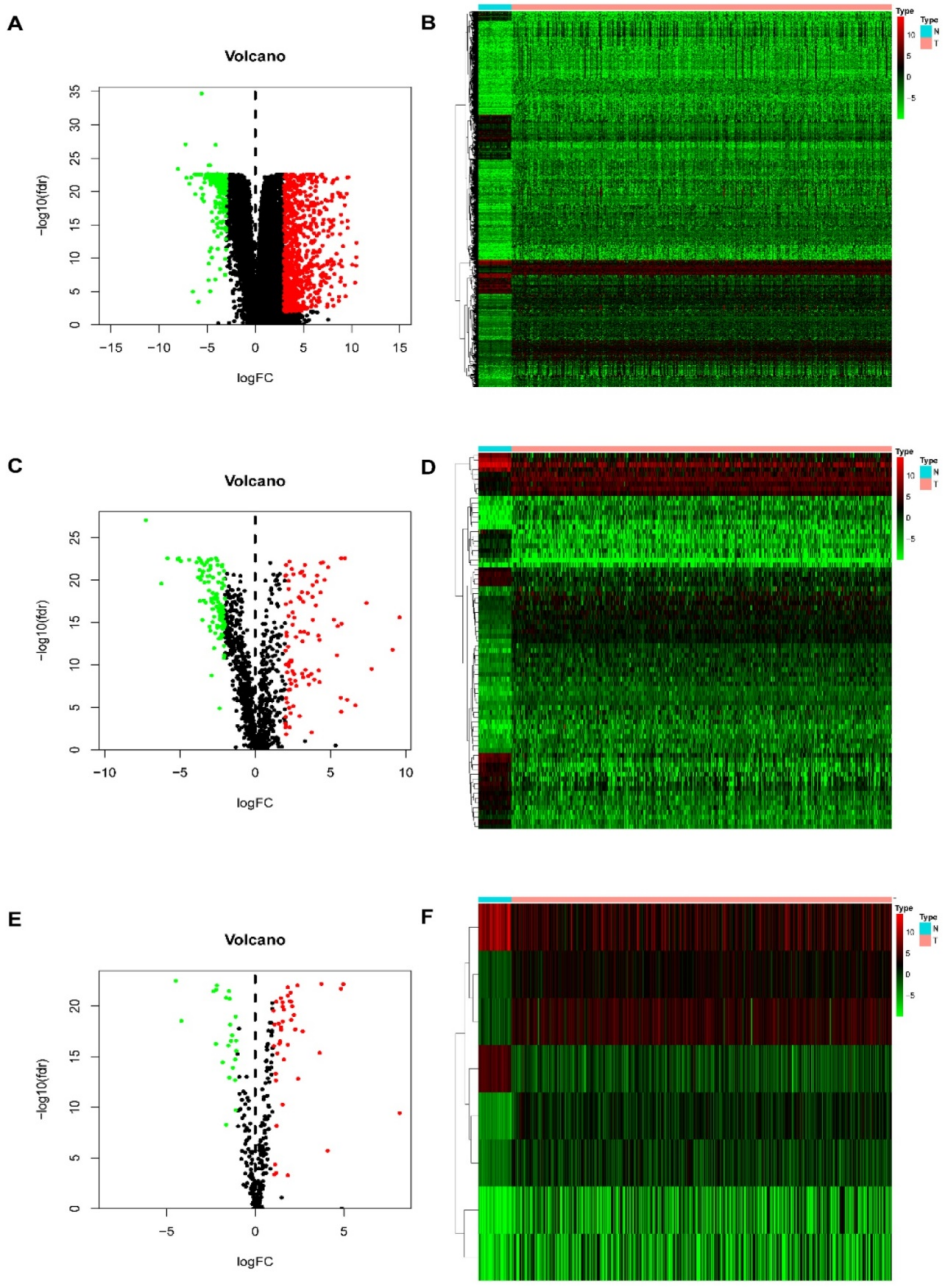
** DEGs, DEIRGs, and DETFs.** (**A**) Volcano plot revealing clusters of upregulated and downregulated DEGs. (**B**) The distinction between DEG expression in tumor and normal tissues revealed by a heatmap. (**C**) Volcano plot demonstrated clusters of upregulated and downregulated DEIRGs. (**D**) Heatmap showing the distinction between the expression of DEIRGs in tumor and normal tissues. (**E**) Volcano plot showing clusters of upregulated and downregulated DETFs. (**F**) Discrimination between DETFs expression in tumor and normal tissues revealed by a heatmap.

In this study, procedures are shown in the **Figure 1** wherein 514 cases were collected, including 473 cases of COAD tissues and 41 cases of non-COAD tissues. The clinical features of the subjects are displayed in **Table 1**. A total of 2450 DEGs (**Table S1**), 79 DEIRGs (**Table 2**), and 8 DETFs (**Table 3**) were identified from the genes extracted from COAD tissue and non-COAD tissue samples based on the cut-off values (|LogFC|>1, FDR <0.05). Among these genes, there were 1765 upregulated DEGs, 685 downregulated DEGs (**Figure 2A, B**), 48 upregulated DEIRGs, 31 downregulated DEIRGs (**Figure 2C, D**), 6 upregulated DETFs, and 2 downregulated DETFs (**Figure 2E, F**).

### GO enrichment and KEGG pathway analysis

**Figure 3:**
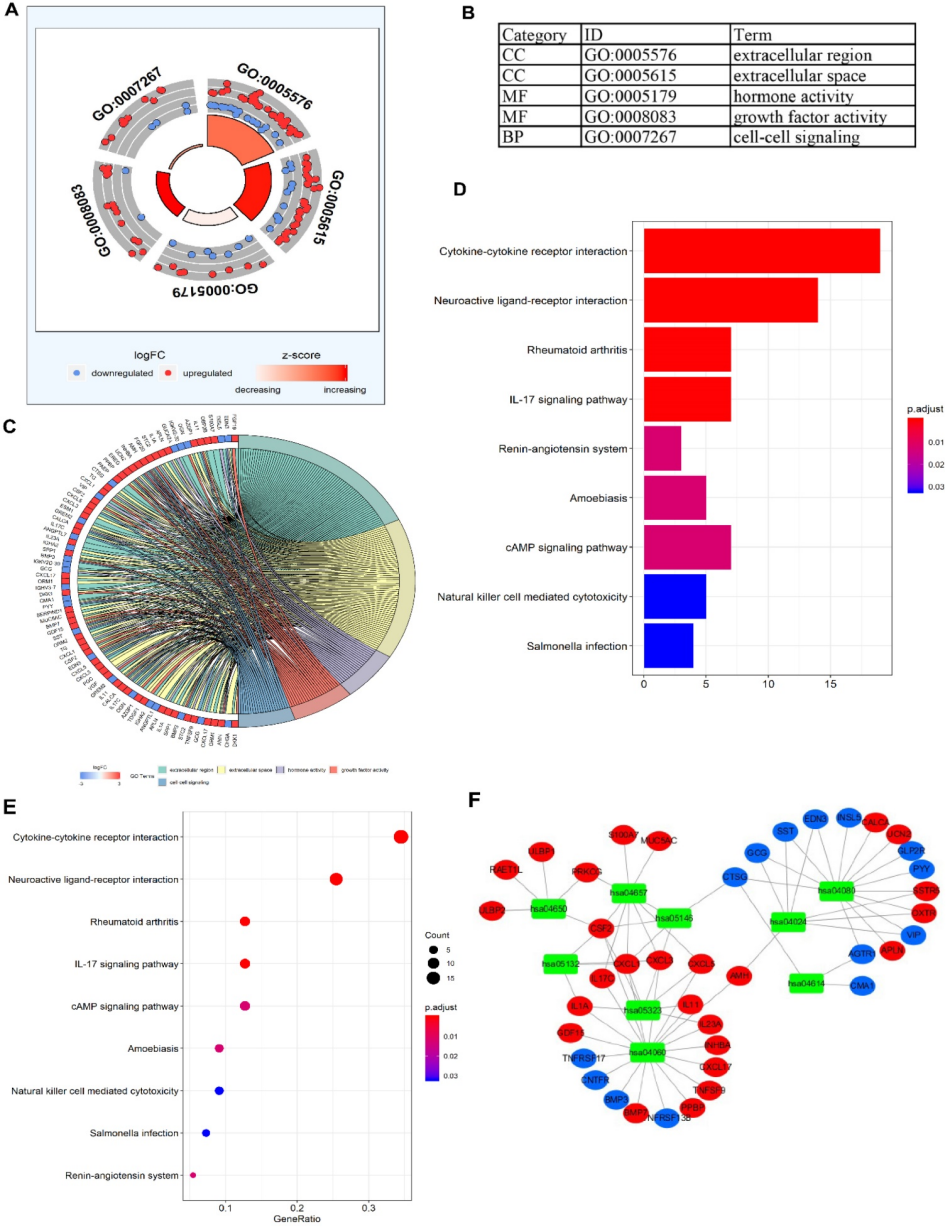
** The functional enrichment analysis of DEIRGs.** (**A**) and (**B**) The outer area of GOchord indicated the expression of DEIRGs, where the maximal aggregation of red dots (upregulated DEIRGs) and blue dots (downregulated DEIRGs) was located on the GO term: extracellular region (GO: 0005576). (**C**) The GOcircle revealed the relationship of 79 DEIRGs with the corresponding GO terms. (**D, E**) The bar plot and bubble plot showed the nine KEGG pathway-enriched DEIRGs. (**F**) KEGG pathways and the corresponding DEIRGs. The green rectangles represented the KEGG pathways, the red ellipses indicated the upregulated DEIRGs, and the blue ellipses indicate the downregulated DEIRGs.

To validate the biological characteristics of DEIRGs, functional relationship analyses were performed, including GO and KEGG pathway analysis. DEIRGs were significantly enriched in five GOs (*P* <0.05), with the most enriched term, “GO: 0005576 extracellular region” in CC category (**Figure 3A, B**). The visual presentation of the association of 79 DEIRGs and relevant GO terms is shown in **Figure 3C**. **Figure 3D** showed the nine KEGG pathways enriched DEIRGs with statistical significance (*P* <0.05). Moreover, it was apparent from **Figure 3E** that the nine KEGG pathways sorted by the number of enriched DEIRGs were in order as follows: Cytokine-cytokine receptor interaction, Neuroactive ligand-receptor interaction, Rheumatoid arthritis, IL-17 signaling pathway, cAMP signaling pathway, Amoebiasis, Natural killer (NK) cell mediated cytotoxicity, Salmonella infection and Renin-angiotensin system (RAS). The nine statistically significant signaling pathways in the KEGG database (*P* <0.05) are shown in **Table 4**, and a network (**Figure 3F**) used for visualizing the interaction between signaling pathways and DEIRGs (*P* <0.05) was constructed. Based on the network visualization, it was evident that hsa04060 (cytokine-cytokine receptor interaction) was frequently used to validate the KEGG pathway.

### TFs-IRGs regulatory network

**Figure 4:**
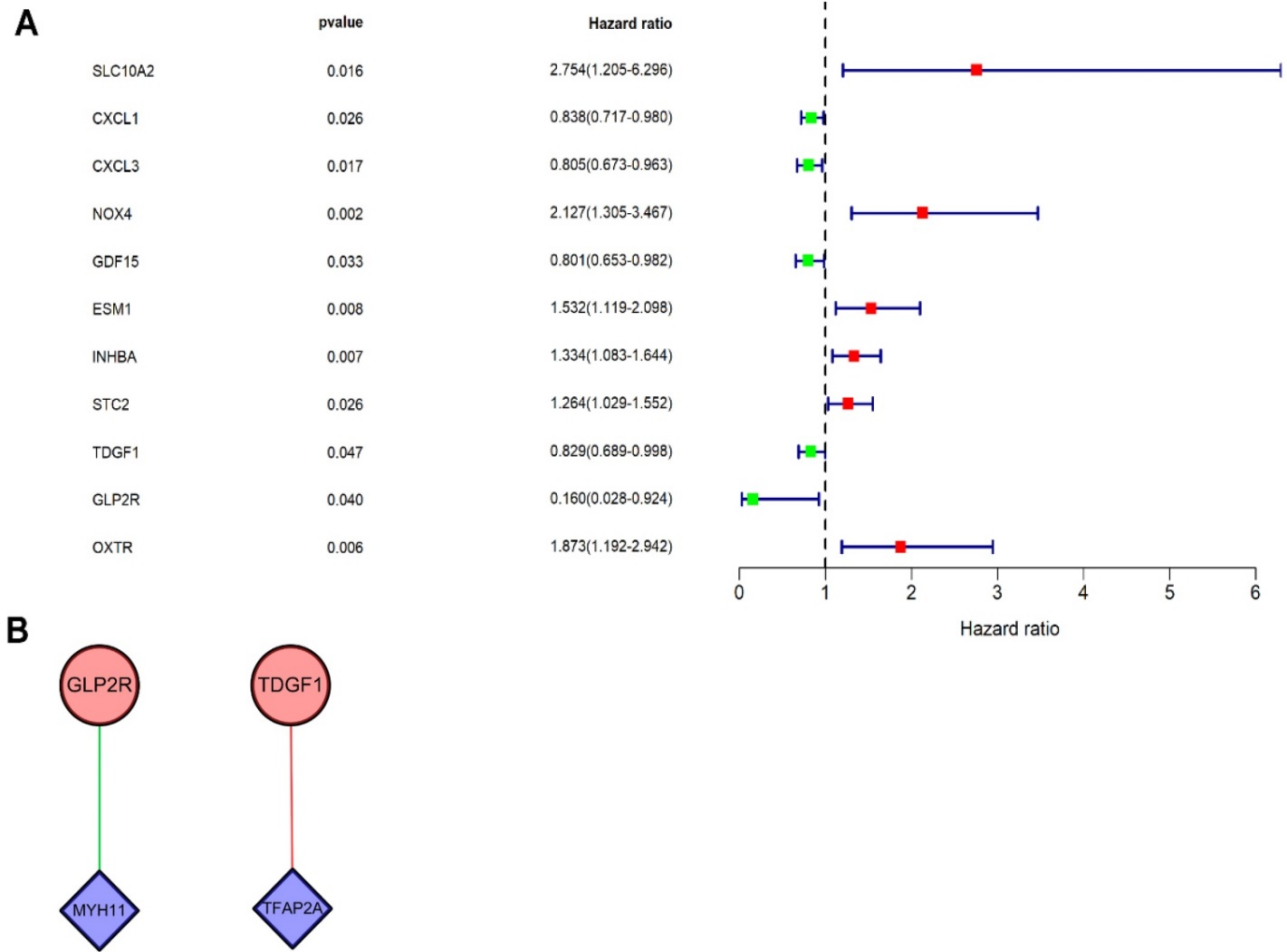
** Univariate Cox regression analysis and TF regulatory network.** (**A**) Forest plot demonstrated the risk classification of 11 prognosis-related IRGs, including six high-risk prognosis-related IRGs (HR>1) and five low-risk prognosis-related IRGs (HR<1). The red squares and green squares indicate the high-risk and low-risk prognosis-related IRGs, respectively. (**B**) The network revealed the relationship of DETFs with prognosis-related IRGs. Purple rhombuses represent DETFs, blue circles represent prognosis-related IRGs, and the red and green lines represent positive and negative correlation, respectively.

Univariate Cox regression analysis was performed on DEIRGs for screening prognosis-related IRGs using “survival package” of R software (*P*<0.05). Consequently, 11 types of genes (**Figure 4A**) were identified, including six high-risk IRGs as well as five low-risk IRGs. Co-expression analysis (Cor filter>0.4 and *P* value filter<0.001) was performed by incorporating seven prognosis-related IRGs and DETFs, which revealed two types of DETFs (TFAP2A and MYH11) and two types of low-risk prognosis-related IRGs (GLP2R and TDGF1) for regulatory network construction (**Figure 4B**). IRG GLP2R was negatively modulated by TF MYH11, whereas IRG TDGF1 was positively modulated by TF TFAP2A.

### Seven-IRG prognostic model

**Figure 5:**
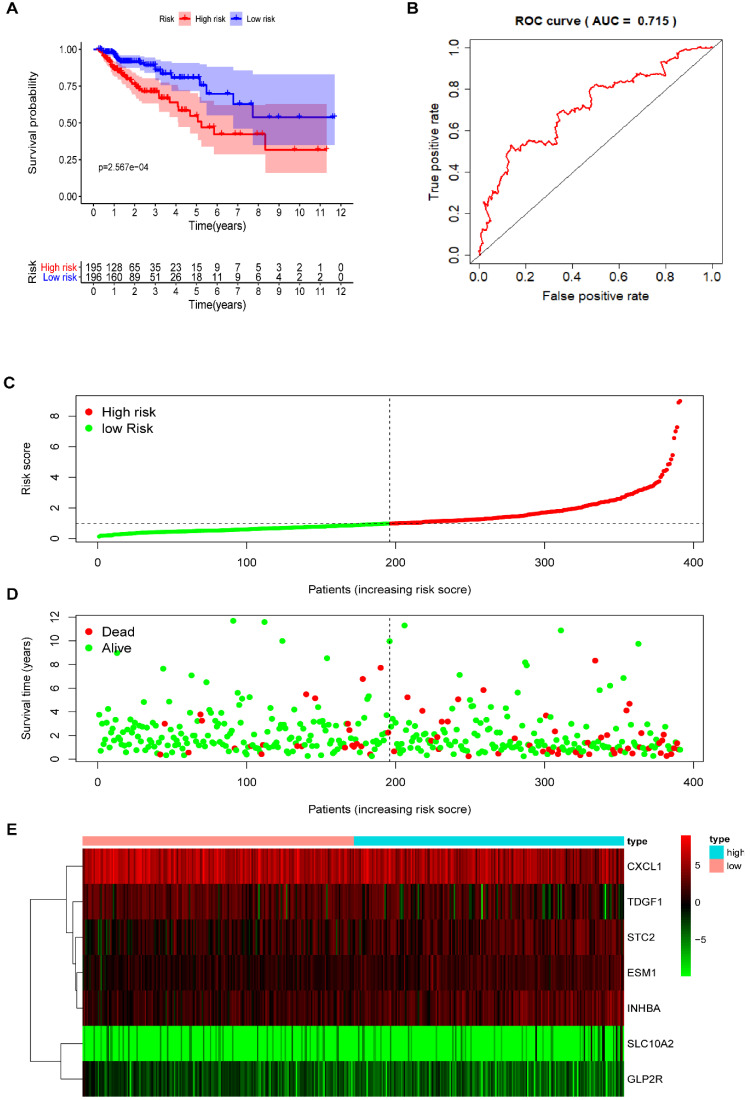
** The prognostic model based on IRGs in COAD.** (**A**) OS curves for patients in high-risk (red curve) and low-risk group (blue curve). (**B**) ROC curve suggesting the feasibility of our prognostic model. (**C**) Patients of high-risk (red dots) and low-risk group (green dots), and the distribution of their corresponding riskScore. (**D**) Patients in high-risk (red dots) and low-risk group (green dots), and their corresponding survival status. (**E**) Discrimination of the expression of seven prognosis-related IRGs between high-risk and low-risk group, as revealed by a heatmap.

Multivariate Cox regression analysis further screened seven prognosis-related IRGs, including four high-risk IRGs and three low-risk IRGs, out of the 11 prognosis-related IRGs identified from univariate Cox regression analysis. The expression of risk coefficients was combined to calculate the riskScore, which was supportive of the prognostic model construction. This riskScore was calculated as the sum of the expression quantities of selected IRGs when multiplied with their corresponding coefficients, as represented by the following formula: riskScore = the expression quantity of SLC10A2*(0.9319) + the expression quantity of CXCL1*(-0.2474) + the expression quantity of ESM1*(0.4490) + the expression quantity of INHBA*(0.2186) + the expression quantity of GLP2R*(-2.1451) + the expression quantity of STC2* (0.1822) + the expression quantity of TDGF1* (-0.2097). The above results of multivariate Cox regression analysis are shown in **Table 5**. Patients were categorized into two groups, namely the high-risk group and the low-risk group, using median riskScore (0.984) as a cut-off value. The Kaplan-Meier (KM) survival curves of both high-risk and low-risk group are displayed in **Figure 5A**, in which the red curve represents the high-risk group (N=195) and the blue curve represents the low-risk group (N=196). And it was clear that the survival rate of patients in the high-risk group was significantly lower than that in the low-risk group (*P* =2.567e-04). The AUC of ROC was approximately 0.715 (**Figure 5B**), and the IRG-based prognostic model was relatively accurate. RiskScore curve, reflecting patient distribution in both high-risk and low-risk groups, are displayed in **Figure 5C**. Similarly, survival status plot, illustrating the survival status of patients in both high-risk and low-risk group, is shown in **Figure 5D**. A heatmap revealing the expression of seven prognosis-related IRGs is shown in **Figure 5E**.

### Independent prognostic analysis

**Figure 6:**
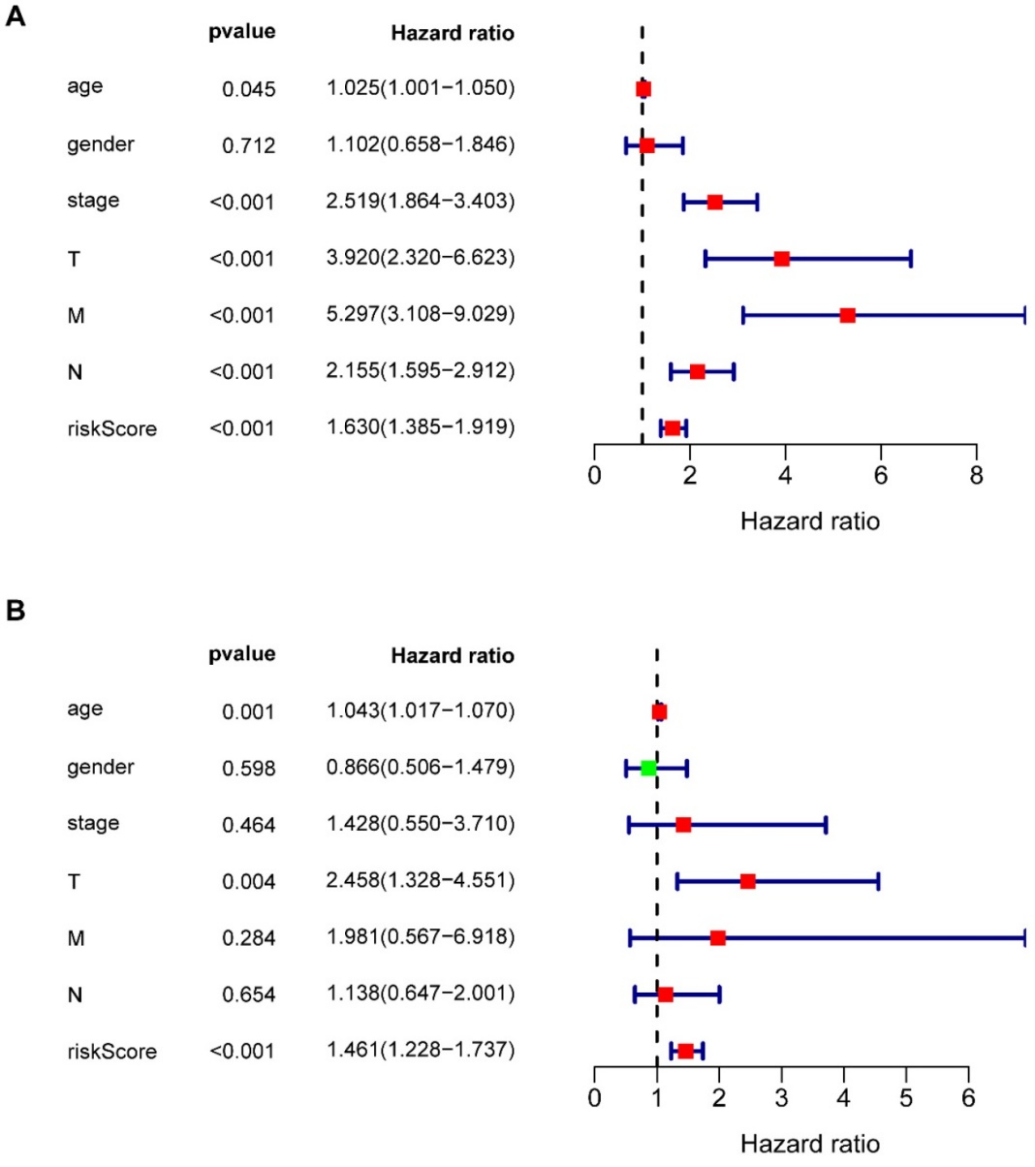
** Univariate and multivariate independent prognostic analysis.** (**A, B**) Forest plots of univariate and multivariate independent prognostic analysis.

Multivariate Cox regression analysis revealed that seven types of prognosis-related IRGs correlated with the prognosis of COAD patients. Univariate independent prognostic analysis (**Figure 6A**) and multivariate independent prognostic analysis (**Figure 6B**) revealed that age, T staging, and riskScore were significantly independent prognostic factors (**Table 6**) (*P* <0.05).

### Seven-IRG prognostic model and clinical features

**Figure 7:**
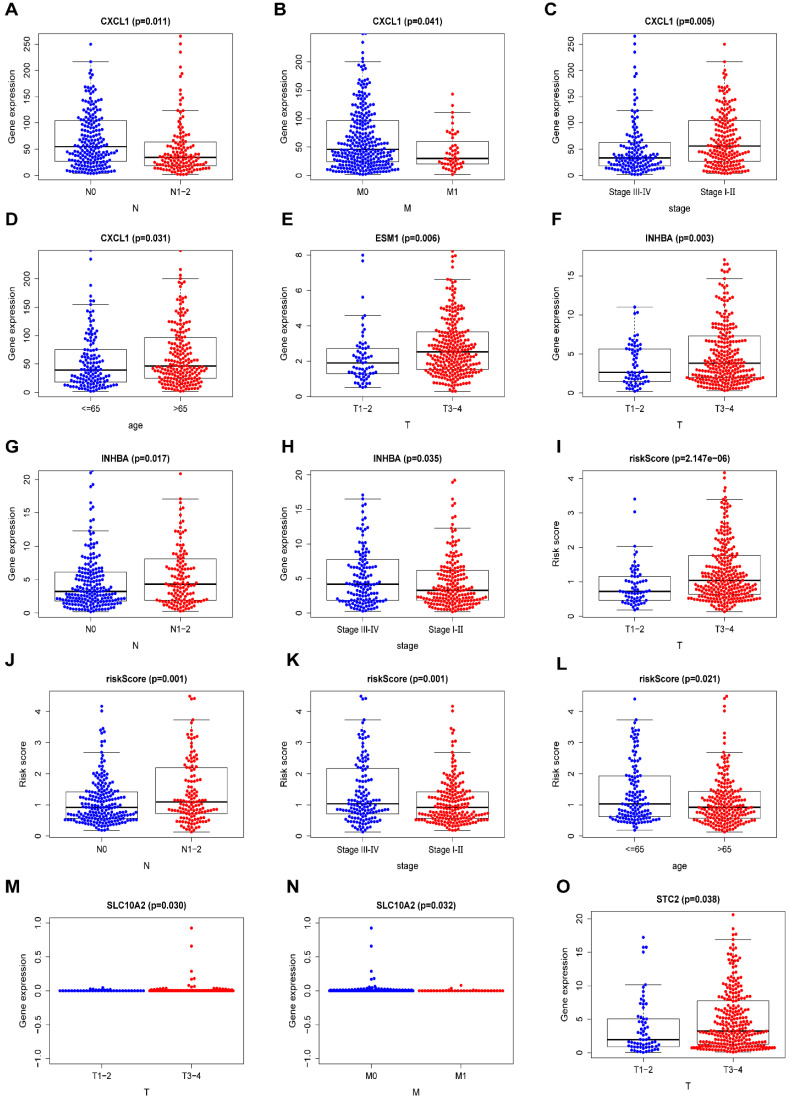
** Comparison of the median expression of seven IRGs based on clinical features.** (**A-D**) A comparison of the median expression of CXCL1 based on N staging, M staging, tumor stage, and age (≤65/>65 years) with statistical significance, respectively. (**E**) A comparison of the median expression of ESM1 in terms of T staging with statistical significance. (**F-H**) A comparison of the median expression of INHBA in terms of T staging, N staging, and tumor stage with statistical significance, respectively. (**I-L**) A comparison of the median riskScore in terms of T staging, N staging, tumor stage, and age (≤65/>65 years) with statistical significance, respectively. (**M, N**) A comparison of the median expression of SLC10A2 in terms of T staging and N staging with statistical significance, respectively. (**O**) A comparison of the median expression of STC2 in terms of T staging with statistical significance.

Some clinical features of COAD, including age, sex, tumor stage, T staging, N staging, and M staging, were assessed for their probable correlation with prognosis-related IRGs via “beeswarm R” packages of R software, as shown in **Table 7** (*P* <0.05). The median values of the selected gene expression were used as cut-off values, and the amounts of medians were found to be directly proportional to the specific clinical features. The median values in T1-2 staging were lower than those in T3-4 staging among ESM1 expression, INHBA expression, STC2 expression and riskScore (**Figure 7E,F,O and I**). In terms of the N staging, the median values of CXCL1 expression were relatively higher in N0 staging than in N1-2 staging (**Figure 7A**). Meanwhile, the median values of INHBA expression and riskScore were relatively higher in N1-2 staging than in N0 staging (**Figure 7G, J**). Moreover, in terms of M staging, the median values of CXCL1 expression were relatively higher in M0 staging than in M1 staging (**Figure 7B**), and for the tumor stage, the median values of INHBA expression and riskScore were significantly elevated in stage Ⅲ-Ⅳ than in stage I-Ⅱ (**Figure 7H, K**). Meanwhile, the median values of CXCL1 expression were higher in stage I-Ⅱ than in stage Ⅲ-Ⅳ (**Figure 7C**). The patient's age also played an important role, and the median values of CXCL1 expression were significantly higher in patients aged >65 years than in patients aged <65 years (**Figure 7D**); the trend was exactly the opposite for riskScore (**Figure 7L**). Additionally, the median values of SLC10A2 expression were also statistically different between T staging and M staging (*P* <0.05) (**Figure 7M, N**).

### Immunocyte infiltration analysis

**Figure 8:**
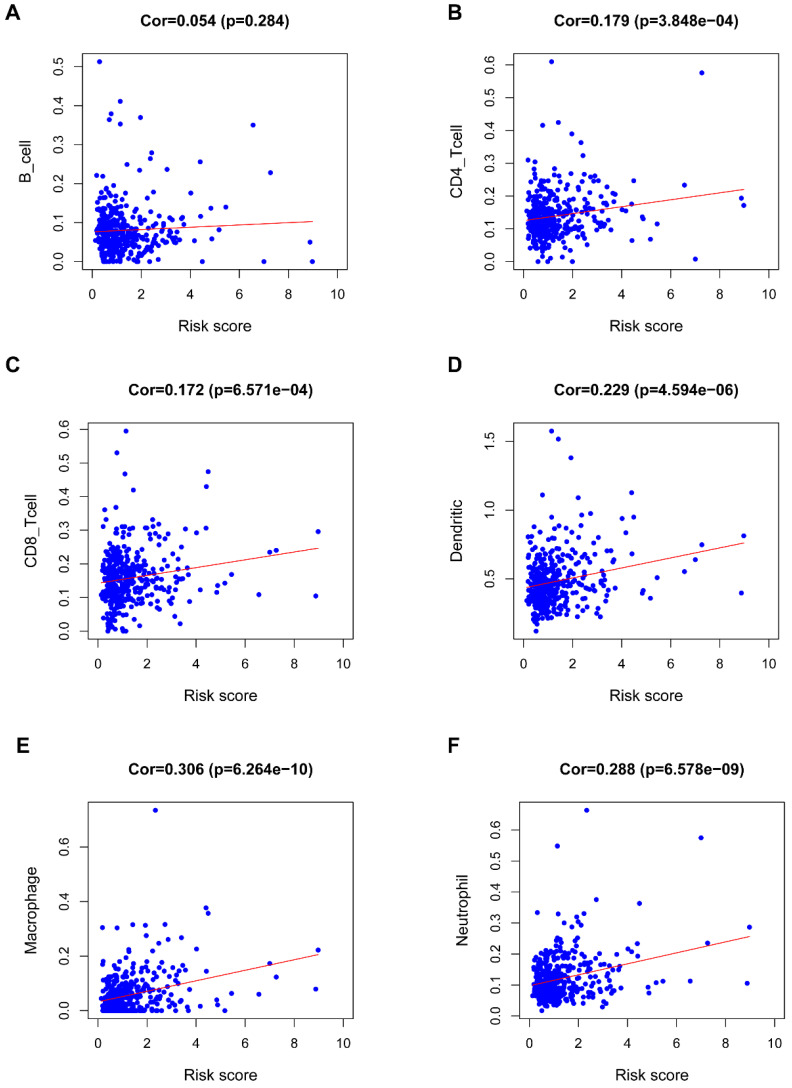
** Correlation of the seven-IRG prognostic model with immunocyte infiltration.** The relationship of the seven-IRG prognostic model with immunocytes, including (**A**) B cell, (**B**) CD4 T cell, (**C**) CD8 T cell, (**D**) Dendritic cell, (**E**) Macrophage, and; (**F**) Neutrophil, was revealed by scatter diagrams.

**Figure 8** revealed a positive correlation of the riskScore of the seven-IRG prognostic model with immunocytes, including dendritic cells, neutrophils, CD8 T cells, macrophages, and CD4 T cells; macrophages were found to have the most significant relationship with riskScore (Cor=0.306, *P*=6.264e-10).

### External verification of seven IRGs using online databases

**Figure 9:**
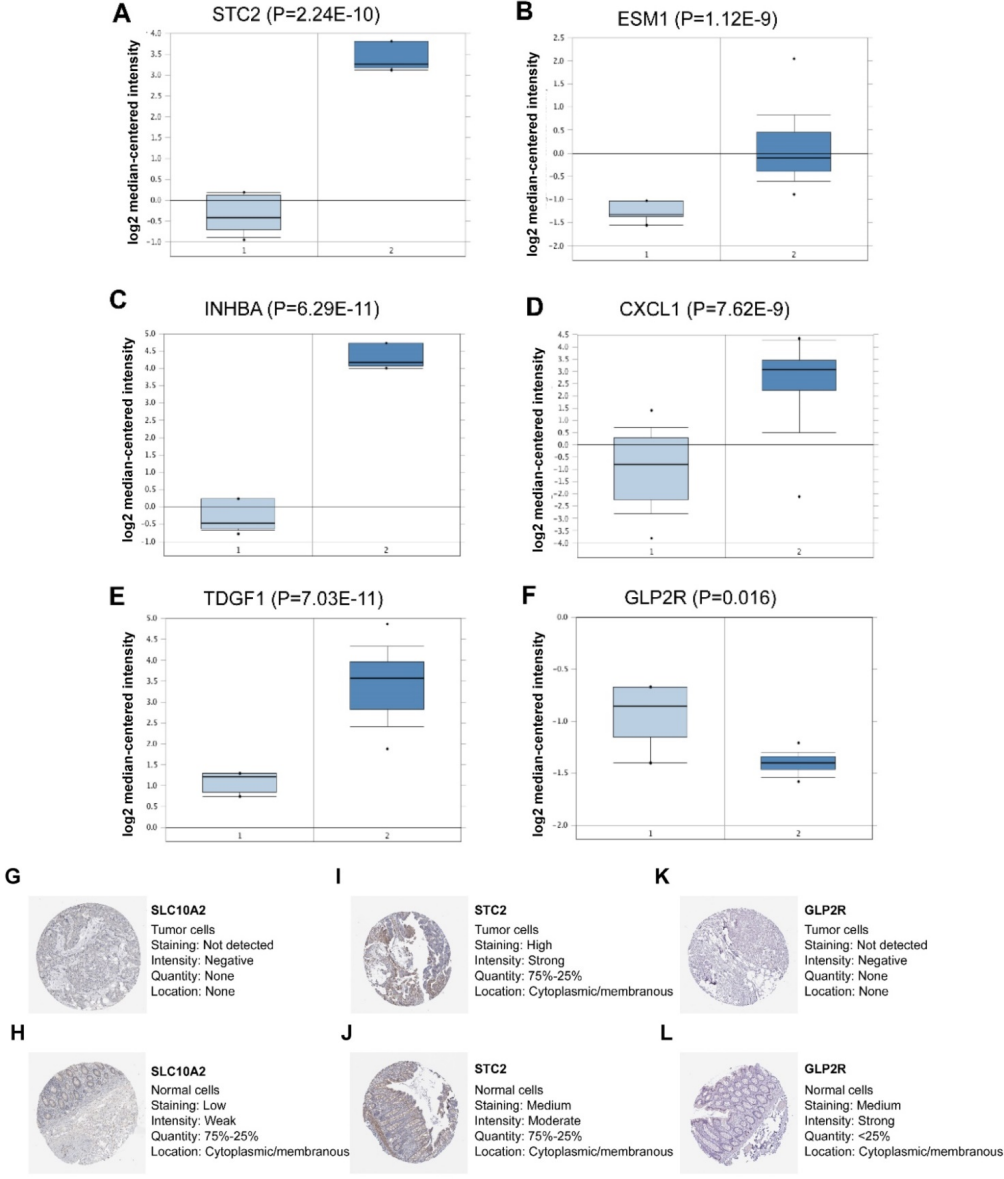
** External verification of IRGs expression in tumor and normal tissues from Oncomine and HPA.** (**A-F**) The expression of six IRGs in tumor and normal tissues with Oncomine, where columns subscript 1 and 2 indicated “tumor tissues” and “normal tissues”, respectively. (**G-L**) The comparison of protein expression of SLC10A2, STC2 and GLP2R between tumor and normal tissues.

**Figure 10:**
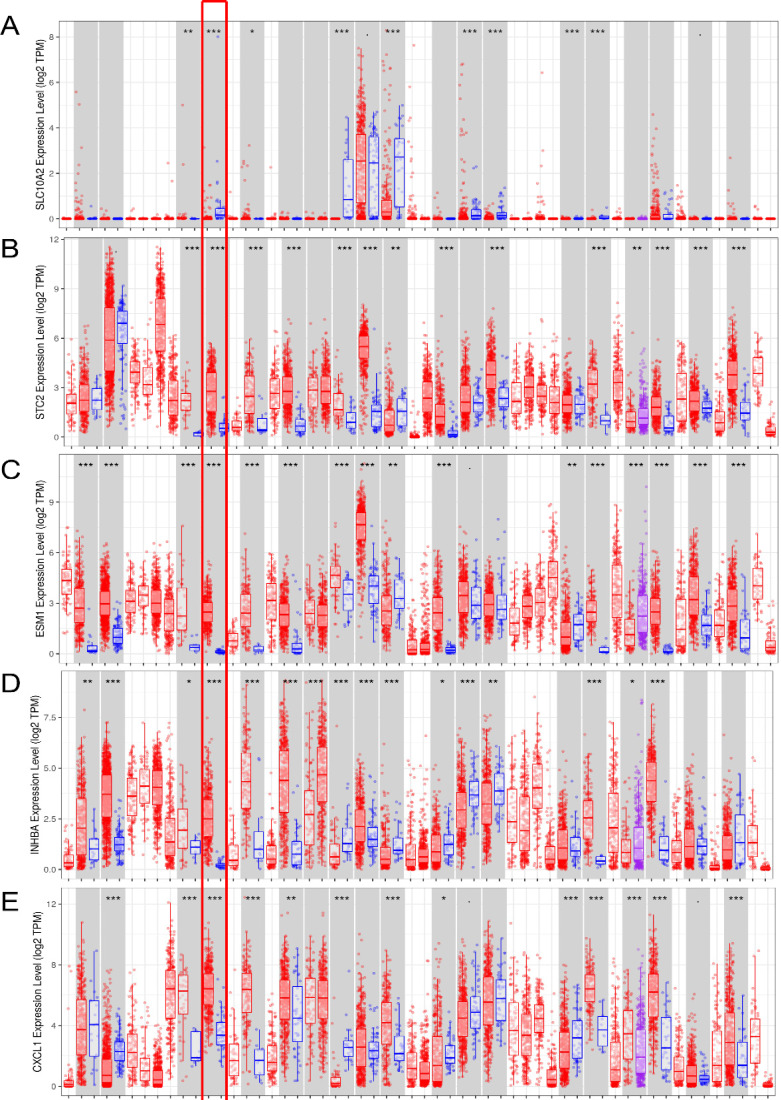

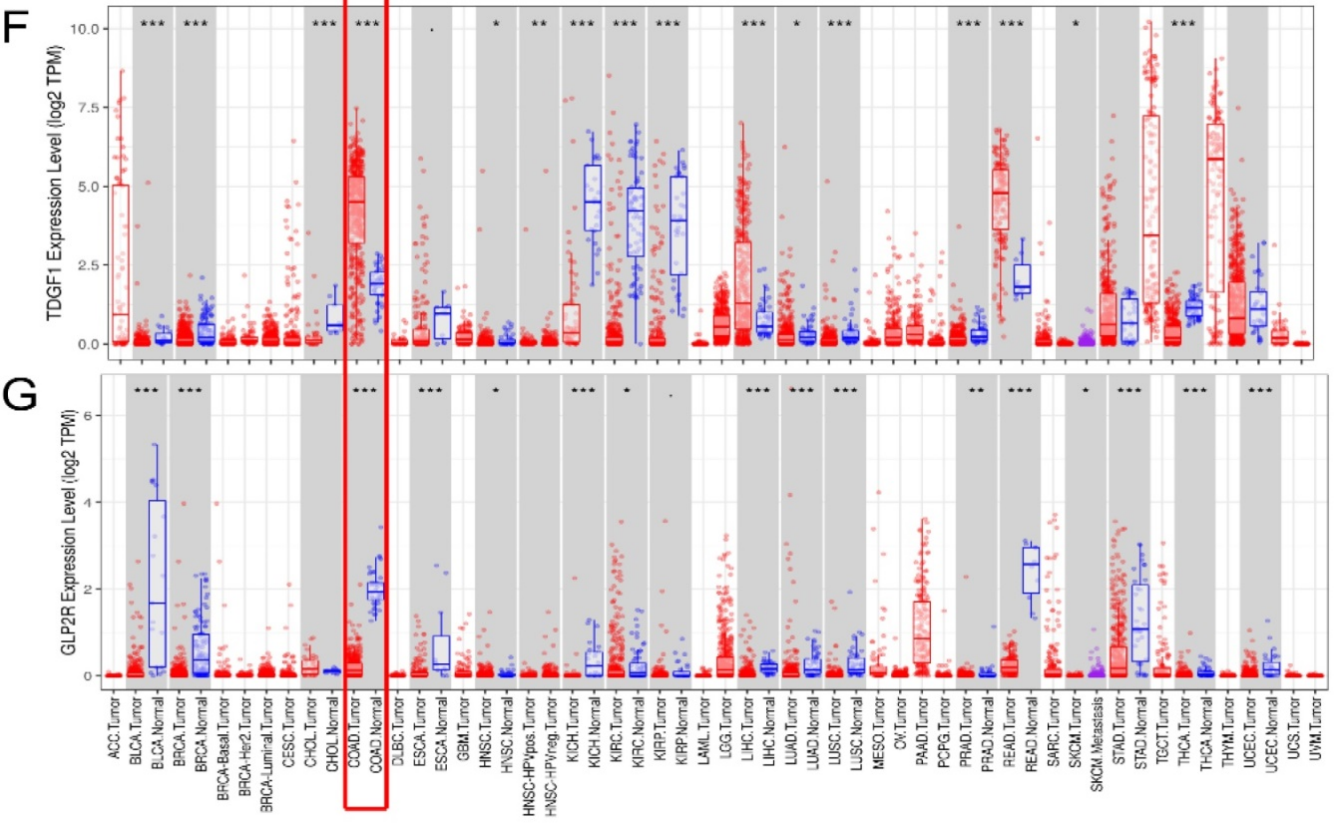
** External verification of IRGs expression based on TIMER.** (**A-G**) The expression of seven IRGs in tumor and normal tissue, where red box plots and blue box plots suggested tumor tissues and normal tissues, respectively. “***” indicated P <0.001.

In the seven-IRG prognostic model, five IRGs were upregulated and the remaining two IRGs were downregulated in COAD. Furthermore, Oncomine was used to externally validate the discrimination regarding the expression of seven IRGs between tumor and normal tissues. As displayed in **Figure 9A-E** and **10B-F**, the expression of STC2, ESM1, INHBA, CXCL1 and TDGF1 was significantly increased in tumor tissues than in normal tissues, whereas GLP2R expression was higher in normal tissues than in tumor tissues (**Figure 9F and 10G**). Similarly, SLC10A2 expression was also increased in normal tissues than in tumor tissues (**Figure 10A**). However, we failed to extract corresponding information on SLC10A2 from Oncomine. The distribution and expression of SLC10A2, STC2, and GLP2R at protein level are shown in **Figure 10G-L**, whereas the distribution and expression of CXCL1, INHBA, ESM1, or TDGF1 remained inaccessible in HPA.

## Discussion

The researches on the predictive effects of biomarkers and their expression on cancer prognosis are a hotspot 24-27. CTHRC1 has been estimated as a peritoneal metastasis-related gene for prognostic prediction in CRC 28, 29. However, it is more dominant to study how the immune-related molecular mechanisms underlying the prognosis of COAD. The formation of the inflammatory environment caused by the deficiency of p53 might become the accelerant of colorectal tumors 30, which may be a direct reference to the investigation, progression and prognosis of COAD related to the immune microenvironment. In addition, some studies have shown that better performance of immunoscore in evaluating high-risk recurrence and metastasis of CRC 31, 32. In this study, we mainly aimed to construct the IRGs-related prognostic model, which were screened out based on immune microenvironment.

By analyzing signaling pathways as well as functional enrichment on DEIRGs, the nine KEGG pathways enriched DEIRGs were shown as follows: Cytokine-cytokine receptor interaction, Neuroactive ligand-receptor interaction, Rheumatoid arthritis, IL-17 signaling pathway, cAMP signaling pathway, Amoebiasis, NK cell mediated cytotoxicity, Salmonella infection and RAS. The above nine KEGG pathways were related to inflammatory response, most of which are validated to have relationship with progression and therapies of colon cancer, including cAMP signaling pathway, Salmonella infection, IL-17 signaling pathway, cytokine-cytokine interaction receptor, NK cell mediated cytotoxicity and RAS. Cytokines along with correlated pathways may be involved in COAD progression in early stage 33. Tseng et al. have shown that the level of IL-17 signaling was enhanced in CRC tissues than normal tissues 34. Additionally, Chin et al. revealed that IL-17 could enhance the DNA binding capacity of NF-κB to stimulate CCR6 expression, potentially involved in the mechanisms of CRC migration 35. cAMP signaling stimulation has been demonstrated to suppress tumor cell migration, including melanoma 36, 37, breast cancer 37 and colon cancer 37, 38 cells. Prospective cohort study has previously demonstrated that high activity of NK cell-mediated cytotoxicity is related to attenuated tumor risk 39, and NK cells have been revealed to be significantly decreased in primary colorectal lesion and liver metastases 40. In a population-based study on patients with Salmonella infection, Mughini-Gras et al. have shown Salmonella infection as a risk factor for low-grade colon tumors 41. To further investigate the mechanisms, Lu et al. have reported that β-catenin signals could be stimulated following Salmonella infection, thereby enhancing colonic tumorigenesis 42. RAS is vitally involved in tumor angiogenesis and tumor cell growth 43, 44. Nakamura et al. have revealed that a combination of RAS inhibitor ARB and anti-PD-L1 antibody showed synergistic anti-tumor effects 44. Herein, in this study, the biological functions of DEIRGs were comprehensively investigated in COAD populations, which could possibly offer promising foundation to illustrate possible molecular regulatory mechanisms.

Multivariate Cox regression analysis revealed seven types of IRGs, including four types of high-risk IRGs (SLC10A2, STC2, ESM1 and INHBA) and three low-risk IRGs (CXCL1, TDGF1 and GLP2R), can be used to construct prognostic model of COAD, which also showed favorable feasibility for the result that AUC being 0.715. Wnt signaling pathway activation, a signal for CRC carcinogenesis, has recently been reported to cause decreased level of SLC10A2 45. STC2 has recently been reported as an independent prognostic biomarker, whose expression is related to colon cancer progression 46. The overexpression of ESM1 in serum of CRC patients has been revealed Ji et al. 47. INHBA expression has been previously reported to be increased in CRC tissues than normal tissues, and high INHBA expression might be used as an independent prognostic factor for lymph node involvement in CRC 48. In terms of CXCL1, increased CXCL1 expression in colon cancer cells has recently been reported, and the carcinogenesis-promoting effect of CXCL2-CXCR2 axis is mediated by Gαi-2 and Gαq/11 49. Miyoshi et al. have demonstrated that CRC patients with high TDGF1 expression following surgery are significantly more likely to suffer palindromia and have poorer DFS in comparison with those with low expression 50. GLP2R is regarded to be a pivotal gene type in CRC progression via the modulation of colonic epithelial integration 51. Both univariate and multivariate independent prognostic analysis showed that age, T staging and riskScore were independent prognostic factors. In this study, seven types of specific IRGs were incorporated to explore the prognostic significance, compared with a previous analogous study. At present, the seven-IRG prognostic model could reflect their correlation with immunocyte infiltration according to the riskScore, which could be used as a type of evaluation indicator of immunologic status. Accumulative studies have demonstrated that immune system components, such as CD8+ and CD45RO+ memory T cells with specific cytokine signatures and possibly B cells, might be prognostic biomarkers, which is associated with tumor evolution with different presence 52-56. Tumor-associated macrophages (TAMs) infiltration has been reported to be associated with OS of patients with prostate cancer and breast cancer 57, 58. Here, notably, riskScore of prognostic model is significantly associated with macrophages, expanding the research vision concerning the relationship of immunocyte with progression and prognosis of COAD. In recent years, the application of nanomedicine based on immune microenvironment, has been identified as a type of novel and individualized anti-cancer therapy. Amphiphilic nanoparticles have been developed as an adjuvant agent, which can be combined with EphA2-derived peptide to assist the immune system to fight against liver metastasis in CRC 59. Kuai et al. have demonstrated that nanodiscs-based phospholipids and apolipoprotein A1 (ApoA1)-mimetic peptides could be used for antigen presentation on dendritic cells to further elevate antitumor immunity 60, and the prognostic model at the center of IRGs might be used as a favorable evaluation of therapeutic efficiency.

A previous research has shown a TFs-miRNA-targeted genes network for exploration of COAD progression 61. To further integrally explore the possible molecular regulatory mechanisms, an IRGs-TFs network was established to elucidate the correlation between MYH11 and TFAP2A, TFs screened out, with IRGs (GLP2R and TDGF1). Nevertheless, studies concerning the regulatory mechanisms underlying TFs and IRGs in COAD are still in progress. Relevant studies have shown that MYH11 can foster the pathogenesis of leukemia and NSCLC 62, 63. Zhang et al. have demonstrated that TFAP2A can enhance anlotinib resistance via the promotion of tumor-triggered angiogenesis in lung tumor cells 64. In our research, IRG GLP2R was positively modulated by TF MYH11, while IRG TDGF1 was negatively modulated by TF TFAP2A. Of note, the TFs-IRGs network could be used to novelly present the prospective molecular mechanisms underlying the tumorigenesis and progression of COAD.

## Strengths and limitations

In this research, IRGs were synthesized for constructing a prognostic model to predict prognosis in COAD patients using bioinformatics. Survival analysis as well as independent prognostic analysis confirmed that the prognostic model was clinically feasible. Additionally, in-depth molecular regulatory correlation analysis, including immunocyte infiltration analysis as well as TFs-IRGs regulatory network, broadened the horizon on researches concerning COAD progression. Multi-database (Oncomine, TIMER and HPA) analysis was utilized to verify the seven prognosis-related IRGs on the basis of RNA expression and protein level. Nevertheless, this study had certain limitations. First, the applicability of the prognostic model needs to be validated for a larger sample population in future studies. Second, we will continue to advance molecular mechanism experiments on the role of IRGs in COAD progression.

## Conclusions

Collectively, DEGs, DEIRGs, and DETFs were retrieved from TCGA, Immport database, and Cistrome database. Univariate and multivariate Cox regression analysis of DEIRGs facilitated the construction of a seven-IRG prognostic model (GLP2R, INHBA CXCL1, STC2, SLC10A2, TDGF1, and ESM1), which could be reliably used for prognostic prediction in COAD patients. Furthermore, both protein and RNA expression of seven IRGs were verified in tumor and normal tissues from Oncomine, TIMER and HPA. In addition, the transcriptional regulatory network and underlying exploration of the association of seven-IRG prognostic model with immunocyte infiltration could potentially uncover new biomolecular interactions in COAD progression, which could be utilized as a potential biomarker for personalized treatment in COAD patients.

## Supplementary Material

Supplementary table S1.

## Figures and Tables

**Table 1:** The clinical features of patients with COAD

Clinical characteristics	Patients (n=452)	Percentage (%)
**Age**		
≤65	185	40.9
>65	267	59.1
**Gender**		
Female	214	47.3
Male	238	52.7
**Stage**		
Stage I	76	16.8
Stage II	178	39.5
Stage III	125	27.7
Stage IV	62	13.7
NA	11	2.43
**T**		
T1+Tis	11	2.43
T2	77	17.1
T3	308	68.2
T4	56	12.4
**N**		
N0	269	59.5
N1	103	22.8
N2	80	17.7
**M**		
M0	334	73.9
M1	62	13.7
MX	49	10.9
NA	7	1.55

**Table 2:** Differentially expressed IRGs

ID	conMean	treatMean	logFC	P-Value	FDR
AZGP1	3.252323	29.96434	3.203704	3.42E-19	3.66E-18
SLC10A2	4.18078	0.027513	-7.24754	8.62E-32	8.36E-28
ULBP2	0.082211	1.858212	4.498442	6.41E-25	8.06E-23
ULBP1	0.019106	0.330626	4.113139	8.43E-23	2.70E-21
RAET1L	0.070562	1.203264	4.091913	6.56E-22	1.48E-20
PPBP	0.172048	95.76959	9.120612	3.84E-13	1.56E-12
CXCL5	0.410911	18.24542	5.472565	4.33E-16	2.62E-15
CXCL1	8.799406	70.87381	3.009775	1.38E-19	1.60E-18
CXCL3	2.828289	24.19823	3.0969	1.82E-20	2.66E-19
SERPIND1	0.044592	0.677743	3.925873	1.93E-09	5.16E-09
S100A7	0.016623	0.873434	5.71547	1.91E-05	3.34E-05
S100A2	0.344744	7.141855	4.372702	7.51E-25	8.85E-23
PAEP	0.007643	5.862431	9.583065	3.33E-17	2.41E-16
MUC5AC	0.197036	3.692445	4.228042	1.45E-10	4.40E-10
NOX4	0.04653	0.521162	3.485506	2.29E-20	3.25E-19
FABP2	25.21012	3.116326	-3.01609	1.05E-22	3.18E-21
OBP2B	0.003181	0.672605	7.724198	9.48E-11	2.93E-10
ORM2	0.054747	0.502928	3.199494	1.03E-09	2.84E-09
ORM1	0.047965	4.868381	6.665315	2.98E-06	5.69E-06
CTSG	6.076998	0.712937	-3.09151	1.39E-23	6.75E-22
GDF15	10.68297	101.8464	3.253009	9.11E-23	2.87E-21
PGC	0.033891	1.264261	5.221267	6.48E-17	4.47E-16
CST4	0.004423	0.741072	7.388496	4.76E-19	4.92E-18
DES	805.2658	71.04126	-3.50274	7.00E-17	4.78E-16
IL1A	0.16444	1.575974	3.260613	2.22E-15	1.22E-14
OLR1	0.150179	2.463243	4.035804	9.36E-19	9.08E-18
CHP2	147.8248	10.00272	-3.88542	5.71E-25	7.80E-23
IGHA2	6606.429	562.3596	-3.55431	1.03E-22	3.16E-21
IGHV3-7	25.42345	1.847598	-3.78244	1.60E-22	4.54E-21
IGKV2-30	27.19268	2.217962	-3.61591	3.36E-22	8.69E-21
IGKV2D-30	3.770542	0.451631	-3.06156	1.06E-20	1.65E-19
IGKV3D-7	2.09134	0.215496	-3.27869	4.15E-19	4.34E-18
IGKV6D-21	17.68264	2.176387	-3.02233	5.03E-16	3.02E-15
CMA1	2.927801	0.250445	-3.54725	7.53E-25	8.85E-23
CXCL17	0.034713	1.507901	5.44094	1.89E-12	7.11E-12
EDN3	24.2212	2.599488	-3.21997	6.55E-25	8.09E-23
AMELX	0.008581	0.455619	5.730477	2.11E-16	1.34E-15
AMH	0.088308	1.694327	4.262017	2.30E-14	1.09E-13
ANGPTL7	1.532729	0.137638	-3.47716	3.07E-23	1.20E-21
APLN	0.333779	3.295931	3.303721	1.74E-24	1.59E-22
BMP3	11.05522	0.331153	-5.06109	3.52E-26	2.52E-23
BMP7	0.779632	7.913799	3.343504	1.21E-10	3.70E-10
CALCA	0.034036	1.742992	5.678372	3.52E-07	7.39E-07
CHGA	92.38148	4.436465	-4.38012	1.52E-25	3.94E-23
CSF2	0.084739	0.962517	3.505718	3.69E-15	1.95E-14
DKK1	0.09323	1.248865	3.743682	0.006743	0.008879
EREG	0.970221	11.56108	3.574819	7.52E-09	1.87E-08
ESM1	0.048119	2.985192	5.955085	2.66E-26	2.52E-23
FGF19	0.105539	1.499664	3.828796	7.08E-17	4.82E-16
FGF20	0.007843	0.539538	6.104136	6.08E-07	1.25E-06
GCG	22.07889	2.549102	-3.11461	1.87E-25	4.53E-23
GREM2	11.43577	0.931	-3.61863	2.08E-25	4.54E-23
GUCA2A	994.9734	27.9527	-5.1536	1.08E-25	3.30E-23
IL11	0.077994	1.911324	4.615068	3.67E-22	9.33E-21
IL17C	0.034196	0.394312	3.52742	7.56E-10	2.11E-09
IL23A	0.360832	3.159239	3.130177	2.15E-23	9.33E-22
INHBA	0.129143	6.724236	5.702328	4.89E-26	2.56E-23
INSL5	44.25744	0.592798	-6.22224	1.23E-21	2.49E-20
OGN	12.08054	1.111031	-3.44271	2.01E-23	8.85E-22
PYY	73.74668	1.308599	-5.81648	5.41E-26	2.62E-23
SPP1	4.504047	82.43657	4.193991	6.75E-15	3.44E-14
SST	26.97694	0.884603	-4.93055	2.96E-25	5.52E-23
STC2	0.350152	4.92824	3.815018	1.99E-21	3.78E-20
TDGF1	0.688909	6.073362	3.140109	3.55E-14	1.64E-13
TG	0.05107	1.040924	4.34925	1.92E-20	2.78E-19
TNFSF9	0.477718	6.685836	3.806876	7.37E-20	9.16E-19
UCN2	0.015551	0.44417	4.835996	4.22E-24	2.99E-22
VGF	0.294619	2.561019	3.119796	3.56E-10	1.03E-09
VIP	32.02157	3.103312	-3.36716	1.80E-24	1.62E-22
AGTR1	1.838394	0.171684	-3.42062	2.15E-19	2.40E-18
ANGPTL1	6.874726	0.669376	-3.36041	8.51E-22	1.82E-20
CNTFR	9.54465	0.738945	-3.69115	1.29E-23	6.39E-22
GLP2R	1.971595	0.147852	-3.73714	2.56E-26	2.52E-23
NR1H4	4.862076	0.381219	-3.67288	2.54E-19	2.79E-18
OXTR	0.073788	0.591929	3.003961	3.60E-23	1.38E-21
SSTR5	0.072985	1.415993	4.278078	4.01E-09	1.03E-08
TNFRSF13B	1.136596	0.128902	-3.14037	1.13E-22	3.39E-21
TNFRSF17	11.74497	1.38808	-3.08088	7.23E-22	1.60E-20
PRKCG	0.058626	0.775783	3.726051	3.87E-10	1.11E-09

**Table 3:** Differentially expressed TFs

ID	conMean	treatMean	logFC	pValue	FDR
CBX2	0.237276	3.144468	3.728177	4.06E-25	6.52E-23
ELF5	0.002937	0.827961	8.139102	1.20E-10	3.66E-10
HOXC11	0.034751	0.590336	4.086414	9.36E-07	1.88E-06
MYH11	282.6829	15.78259	-4.16278	2.00E-20	2.88E-19
PDX1	0.286565	8.182502	4.835606	2.31E-24	1.96E-22
SALL4	0.033764	1.057694	4.969275	4.71E-25	6.86E-23
SPIB	13.82464	0.621519	-4.4753	8.16E-26	3.18E-23
TFAP2A	0.078334	0.973795	3.635912	5.98E-17	4.14E-16

**Table 4:** KEGG pathways enriched differentially expressed IRGs

ID	Description	*p* value	*p*-adjust	*q* value	Count
hsa04060	Cytokine-cytokine receptor interaction	2.96E-14	3.87E-12	3.46E-12	19
hsa04080	Neuroactive ligand-receptor interaction	4.67E-08	3.06E-06	2.73E-06	14
hsa05323	Rheumatoid arthritis	3.08E-06	0.000108	9.66E-05	7
hsa04657	IL-17 signaling pathway	3.31E-06	0.000108	9.66E-05	7
hsa04614	Renin-angiotensin system	0.000504	0.012465	0.011118	3
hsa05146	Amoebiasis	0.000661	0.012465	0.011118	5
hsa04024	cAMP signaling pathway	0.000666	0.012465	0.011118	7
hsa04650	Natural killer cell mediated cytotoxicity	0.002028	0.032037	0.028574	5
hsa05132	Salmonella infection	0.002201	0.032037	0.028574	4

**Table 5:** Seven types of prognosis-related IRGs

IRGs	coef	exp(coef)	se(coef)	*z*	*p*
SLC10A2	0.9319	2.5393	0.5051	1.84	0.0650
CXCL1	-0.2474	0.7808	0.0812	-3.05	0.0023
ESM1	0.4490	1.5667	0.1818	2.47	0.0135
INHBA	0.2186	1.2443	0.1102	1.98	0.0472
STC2	0.1822	1.1998	0.1108	1.64	0.1001
TDGF1	-0.2097	0.8108	0.1039	-2.02	0.0435
GLP2R	-2.1451	0.1171	0.9216	-2.33	0.0199

**Table 6:** Univariate and multivariate independent prognostic analysis

Variables	Univariate analysis		Multivariate analysis	
	Hazard ratio (95% CI)	*p* value	Hazard ratio (95% CI)	*p* value
age	1.025 (1.001-1.050)	0.045	1.043 (1.107-1.070)	0.001
gender	1.102 (0.667-1.494)	0.712	0.866 (0.506-1.479)	0.598
stage	2.519 (1.864-3.403)	<0.001	1.428 (0.550-3.710)	0.464
T	3.920 (2.320-6.623)	<0.001	2.458 (1.328-4.551)	0.004
M	5.297 (3.108-9.029)	<0.001	1.981 (0.567-6.918)	0.284
N	2.155 (1.595- 2.912)	<0.001	1.138 (0.647-2.001)	0.654
riskScore	1.630 (1.385- 1.919)	<0.001	1.461 (1.228-1.737)	<0.001

**Table 7:** The clinical correlation analysis

Gene	Age (≤65/>65)		Gender (male/female)		Stage(I-II/III-IV)		T(T1-T2/T3-T4)		M(M0/M1)		N(N0/N1-N3)	
	*t*	*P*	*t*	*P*	*t*	*P*	*t*	*P*	*t*	*P*	*t*	*P*
SLC10A2	1.241	0.216	1.814	0.072	0.547	0.585	-2.178	0.030	2.149	0.032	-0.603	0.547
CXCL1	-2.168	0.031	-0.508	0.612	-2.822	0.005	0.897	0.372	2.084	0.041	2.555	0.011
ESM1	1.653	0.099	-0.506	0.613	1.915	0.057	-2.778	0.006	-1.718	0.091	1.838	0.067
INHBA	1.837	0.067	0.616	0.538	2.215	0.035	-3.034	0.003	-1.072	0.288	-2.409	0.017
STC2	0.502	0.616	-0.767	0.444	0.918	0.360	-2.103	0.038	-0.051	0.960	-1.07	0.286
TDGF1	0.484	0.629	-1.066	0.287	0.381	0.703	-1.705	0.284	-0.954	0.343	-0.4	0.690
GLP2R	0.337	0.736	0.94	0.348	1.277	0.203	-0.06	0.953	0.515	0.608	-1.374	0.171
riskScore	2.323	0.021	0.571	0.568	3.279	0.001	-4.898	2.147e-06	-1.662	0.102	-3.298	0.001

## Abbreviations

COAD: Colon adenocarcinoma
DEGs: Differentially expressed genes
DEIRGs: Differentially expressed immune-related genes
DETFs: Differentially expressed transcription factors
TCGA: The Cancer Genome Atlas Project
IMMPORT: Immunology Database and Analysis Portal
TIMER: Tumor Immune Estimation Resource
GO: Gene ontology
KEGG: Kyoto Encyclopedia of Genes and Genomes
DAVID: The Database for Annotation, Visualization and Integrated Discovery
AUC: Area under the curve
ROC: Receiver operating characteristic
FDR: False discovery rate

## References

[1] Fidler MM, Soerjomataram I, Bray F (2016). A global view on cancer incidence and national levels of the human development index. Int J Cancer.

[2] Arnold M, Sierra MS, Laversanne M, Soerjomataram I, Jemal A, Bray F (2017). Global patterns and trends in colorectal cancer incidence and mortality. Gut.

[3] Mutch MG (2007). Molecular profiling and risk stratification of adenocarcinoma of the colon. J Surg Oncol.

[4] Bray F, Ferlay J, Soerjomataram I, Siegel RL, Torre LA, Jemal A (2018). Global cancer statistics 2018: GLOBOCAN estimates of incidence and mortality worldwide for 36 cancers in 185 countries. CA Cancer J Clin.

[5] Muro K (2017). Systemic chemotherapy for metastatic colorectal cancer -Japanese Society for Cancer of the Colon and Rectum (JSCCR) Guidelines 2016 for treatment of colorectal cancer. Nihon Shokakibyo Gakkai Zasshi.

[6] Harada H, Taniguchi T, Tanaka N (1998). The role of interferon regulatory factors in the interferon system and cell growth control. Biochimie.

[7] Gough DJ, Levy DE, Johnstone RW, Clarke CJ (2008). IFNgamma signaling-does it mean JAK-STAT?. Cytokine Growth Factor Rev.

[8] Slattery ML, Lundgreen A, Bondurant KL, Wolff RK (2011). Interferon-signaling pathway: associations with colon and rectal cancer risk and subsequent survival. Carcinogenesis.

[9] Barber DL, Wherry EJ, Masopust D, Zhu B, Allison JP, Sharpe AH (2006). Restoring function in exhausted CD8 T cells during chronic viral infection. Nature.

[10] Salem M, Wallace C, Velegraki M, Li A, Ansa-Addo E, Metelli A (2019). GARP Dampens Cancer Immunity by Sustaining Function and Accumulation of Regulatory T Cells in the Colon. Cancer Res.

[11] Dalerba P, Sahoo D, Paik S, Guo X, Yothers G, Song N (2016). CDX2 as a Prognostic Biomarker in Stage II and Stage III Colon Cancer. N Engl J Med.

[12] Li B, Cui Y, Diehn M, Li R (2017). Development and Validation of an Individualized Immune Prognostic Signature in Early-Stage Nonsquamous Non-Small Cell Lung Cancer. JAMA Oncol.

[13] Lin P, Guo YN, Shi L, Li XJ, Yang H, He Y (2019). Development of a prognostic index based on an immunogenomic landscape analysis of papillary thyroid cancer. Aging (Albany NY).

[14] Bhattacharya S, Dunn P, Thomas CG, Smith B, Schaefer H, Chen J (2018). ImmPort, toward repurposing of open access immunological assay data for translational and clinical research. Sci Data.

[15] Ritchie ME, Phipson B, Wu D, Hu Y, Law CW, Shi W (2015). limma powers differential expression analyses for RNA-sequencing and microarray studies. Nucleic Acids Res.

[16] Walter W, Sanchez-Cabo F, Ricote M (2015). GOplot: an R package for visually combining expression data with functional analysis. Bioinformatics.

[17] Mei S, Meyer CA, Zheng R, Qin Q, Wu Q, Jiang P (2017). Cistrome Cancer: A Web Resource for Integrative Gene Regulation Modeling in Cancer. Cancer Res.

[18] Liang J, Cui Y, Meng Y, Li X, Wang X, Liu W (2019). Integrated analysis of transcription factors and targets co-expression profiles reveals reduced correlation between transcription factors and target genes in cancer. Funct Integr Genomics.

[19] Lin P, Guo Y-N, Shi L, Li X-J, Yang H, He Y (2019). Development of a prognostic index based on an immunogenomic landscape analysis of papillary thyroid cancer. Aging.

[20] Li T, Fan J, Wang B, Traugh N, Chen Q, Liu JS (2017). TIMER: A Web Server for Comprehensive Analysis of Tumor-Infiltrating Immune Cells. Cancer Res.

[21] Rhodes DR, Kalyana-Sundaram S, Mahavisno V, Varambally R, Yu J, Briggs BB (2007). Oncomine 3.0: genes, pathways, and networks in a collection of 18,000 cancer gene expression profiles. Neoplasia.

[22] Liu G-M, Xie W-X, Zhang C-Y, Xu J-W (2020). Identification of a four-gene metabolic signature predicting overall survival for hepatocellular carcinoma. Journal of cellular physiology.

[23] Pontén F, Schwenk JM, Asplund A, Edqvist PHD (2011). The Human Protein Atlas as a proteomic resource for biomarker discovery. J Intern Med.

[24] Rosenwald A, Wright G, Wiestner A, Chan WC, Connors JM, Campo E (2003). The proliferation gene expression signature is a quantitative integrator of oncogenic events that predicts survival in mantle cell lymphoma. Cancer cell.

[25] Sotiriou C, Wirapati P, Loi S, Harris A, Fox S, Smeds J (2006). Gene expression profiling in breast cancer: understanding the molecular basis of histologic grade to improve prognosis. J Natl Cancer Inst.

[26] Qu L, Wang ZL, Chen Q, Li YM, He HW, Hsieh JJ (2018). Prognostic Value of a Long Non-coding RNA Signature in Localized Clear Cell Renal Cell Carcinoma. Eur Urol.

[27] Whitfield ML, Sherlock G, Saldanha AJ, Murray JI, Ball CA, Alexander KE (2002). Identification of genes periodically expressed in the human cell cycle and their expression in tumors. Mol Biol Cell.

[28] Tan F, Liu F, Liu H, Hu Y, Liu D, Li G (2013). CTHRC1 is associated with peritoneal carcinomatosis in colorectal cancer: a new predictor for prognosis. Med Oncol.

[29] Wan Q, Tang J, Han Y, Wang D (2018). Co-expression modules construction by WGCNA and identify potential prognostic markers of uveal melanoma. Exp Eye Res.

[30] Schwitalla S, Ziegler PK, Horst D, Becker V, Kerle I, Begus-Nahrmann Y (2013). Loss of p53 in enterocytes generates an inflammatory microenvironment enabling invasion and lymph node metastasis of carcinogen-induced colorectal tumors. Cancer Cell.

[31] Mlecnik B, Bindea G, Angell HK, Maby P, Angelova M, Tougeron D (2016). Integrative Analyses of Colorectal Cancer Show Immunoscore Is a Stronger Predictor of Patient Survival Than Microsatellite Instability. Immunity.

[32] Mlecnik B, Bindea G, Kirilovsky A, Angell HK, Obenauf AC, Tosolini M (2016). The tumor microenvironment and Immunoscore are critical determinants of dissemination to distant metastasis. Sci Transl Med.

[33] Saadi A, Shannon NB, Lao-Sirieix P, O'Donovan M, Walker E, Clemons NJ (2010). Stromal genes discriminate preinvasive from invasive disease, predict outcome, and highlight inflammatory pathways in digestive cancers. Proc Natl Acad Sci U S A.

[34] Tseng J-Y, Yang C-Y, Liang S-C, Liu R-S, Yang S-H, Lin J-K (2014). Interleukin-17A modulates circulating tumor cells in tumor draining vein of colorectal cancers and affects metastases. Clin Cancer Res.

[35] Chin C-C, Chen C-N, Kuo H-C, Shi C-S, Hsieh MC, Kuo Y-H (2015). Interleukin-17 induces CC chemokine receptor 6 expression and cell migration in colorectal cancer cells. Journal of cellular physiology.

[36] Dua P, Gude RP (2008). Pentoxifylline impedes migration in B16F10 melanoma by modulating Rho GTPase activity and actin organisation. European journal of cancer (Oxford, England: 1990).

[37] Dong H, Claffey KP, Brocke S, Epstein PM (2015). Inhibition of breast cancer cell migration by activation of cAMP signaling. Breast Cancer Res Treat.

[38] Murata K, Sudo T, Kameyama M, Fukuoka H, Muka M, Doki Y (2000). Cyclic AMP specific phosphodiesterase activity and colon cancer cell motility. Clin Exp Metastasis.

[39] Imai K, Matsuyama S, Miyake S, Suga K, Nakachi K (2000). Natural cytotoxic activity of peripheral-blood lymphocytes and cancer incidence: an 11-year follow-up study of a general population. Lancet.

[40] Halama N, Braun M, Kahlert C, Spille A, Quack C, Rahbari N (2011). Natural killer cells are scarce in colorectal carcinoma tissue despite high levels of chemokines and cytokines. Clin Cancer Res.

[41] Mughini-Gras L, Schaapveld M, Kramers J, Mooij S, Neefjes-Borst EA, Pelt Wv (2018). Increased colon cancer risk after severe Salmonella infection. PLoS ONE.

[42] Lu R, Wu S, Zhang YG, Xia Y, Liu X, Zheng Y (2014). Enteric bacterial protein AvrA promotes colonic tumorigenesis and activates colonic beta-catenin signaling pathway. Oncogenesis.

[43] Egami K, Murohara T, Shimada T, Sasaki K-I, Shintani S, Sugaya T (2003). Role of host angiotensin II type 1 receptor in tumor angiogenesis and growth. J Clin Invest.

[44] Nakamura K, Yaguchi T, Ohmura G, Kobayashi A, Kawamura N, Iwata T (2018). Involvement of local renin-angiotensin system in immunosuppression of tumor microenvironment. Cancer Sci.

[45] Aymeric L, Donnadieu F, Mulet C, du Merle L, Nigro G, Saffarian A (2018). Colorectal cancer specific conditions promote Streptococcus gallolyticus gut colonization. Proc Natl Acad Sci U S A.

[46] Zhang C, Chen S, Ma X, Yang Q, Su F, Shu X (2019). Upregulation of STC2 in colorectal cancer and its clinicopathological significance. Onco Targets Ther.

[47] Ji NY, Kim Y-H, Jang YJ, Kang YH, Lee CI, Kim JW (2010). Identification of endothelial cell-specific molecule-1 as a potential serum marker for colorectal cancer. Cancer Sci.

[48] Okano M, Yamamoto H, Ohkuma H, Kano Y, Kim H, Nishikawa S (2013). Significance of INHBA expression in human colorectal cancer. Oncol Rep.

[49] Chen M-C, Baskaran R, Lee N-H, Hsu H-H, Ho T-J, Tu C-C (2019). CXCL2/CXCR2 axis induces cancer stem cell characteristics in CPT-11-resistant LoVo colon cancer cells via Gαi-2 and Gαq/11. Journal of cellular physiology.

[50] Miyoshi N, Ishii H, Mimori K, Sekimoto M, Doki Y, Mori M (2010). TDGF1 is a novel predictive marker for metachronous metastasis of colorectal cancer. International journal of oncology.

[51] Lu Y, Kweon SS, Tanikawa C, Jia WH, Xiang YB, Cai Q (2019). Large-Scale Genome-Wide Association Study of East Asians Identifies Loci Associated With Risk for Colorectal Cancer. Gastroenterology.

[52] Jochems C, Schlom J (2011). Tumor-infiltrating immune cells and prognosis: the potential link between conventional cancer therapy and immunity. Exp Biol Med (Maywood).

[53] Galon J, Costes A, Sanchez-Cabo F, Kirilovsky A, Mlecnik B, Lagorce-Pagès C (2006). Type, density, and location of immune cells within human colorectal tumors predict clinical outcome. Science.

[54] Fridman WH, Pages F, Sautes-Fridman C, Galon J (2012). The immune contexture in human tumours: impact on clinical outcome. Nat Rev Cancer.

[55] Schmidt M, Hellwig B, Hammad S, Othman A, Lohr M, Chen Z (2012). A comprehensive analysis of human gene expression profiles identifies stromal immunoglobulin κ C as a compatible prognostic marker in human solid tumors. Clin Cancer Res.

[56] Nielsen JS, Sahota RA, Milne K, Kost SE, Nesslinger NJ, Watson PH (2012). CD20+ tumor-infiltrating lymphocytes have an atypical CD27- memory phenotype and together with CD8+ T cells promote favorable prognosis in ovarian cancer. Clin Cancer Res.

[57] Tsutsui S, Yasuda K, Suzuki K, Tahara K, Higashi H, Era S (2005). Macrophage infiltration and its prognostic implications in breast cancer: the relationship with VEGF expression and microvessel density. Oncology reports.

[58] Lissbrant IF, Stattin P, Wikstrom P, Damber JE, Egevad L, Bergh A (2000). Tumor associated macrophages in human prostate cancer: relation to clinicopathological variables and survival. International journal of oncology.

[59] Yamaguchi S, Tatsumi T, Takehara T, Sasakawa A, Yamamoto M, Kohga K (2010). EphA2-derived peptide vaccine with amphiphilic poly(gamma-glutamic acid) nanoparticles elicits an anti-tumor effect against mouse liver tumor. Cancer Immunol Immunother.

[60] Kuai R, Ochyl LJ, Bahjat KS, Schwendeman A, Moon JJ (2017). Designer vaccine nanodiscs for personalized cancer immunotherapy. Nat Mater.

[61] Wei S, Chen J, Huang Y, Sun Q, Wang H, Liang X (2020). Identification of hub genes and construction of transcriptional regulatory network for the progression of colon adenocarcinoma hub genes and TF regulatory network of colon adenocarcinoma. J Cell Physiol.

[62] Clayton EA, Rishishwar L, Huang TC, Gulati S, Ban D, McDonald JF (2020). An atlas of transposable element-derived alternative splicing in cancer. Philos Trans R Soc Lond B Biol Sci.

[63] Ma Q, Xu Y, Liao H, Cai Y, Xu L, Xiao D (2019). Identification and validation of key genes associated with non-small-cell lung cancer. J Cell Physiol.

[64] Zhang L-l, Lu J, Liu R-Q, Hu M-J, Zhao Y-M, Tan S (2020). Chromatin accessibility analysis reveals that TFAP2A promotes angiogenesis in acquired resistance to anlotinib in lung cancer cells. Acta Pharmacol Sin.

